# Hypovolaemia was associated with clustering of major cardiovascular risk factors in general population

**DOI:** 10.1186/1471-2261-14-151

**Published:** 2014-10-31

**Authors:** Xianglei Kong, Xiaojing Ma, Jing Yao, Shuting Zheng, Meiyu Cui, Dongmei Xu

**Affiliations:** Department of Nephrology, Qianfoshan Hospital, Shandong University, No.16766, Jingshi Road, Jinan, 250014 PR China; Department of Physical Examination Center, Qianfoshan Hospital, Shandong University, No.16766, Jingshi Road, Jinan, China; The Blood Purification Center, Shandong Veterans General Hospital, No. 23, Jiefang Road, Jinan, China; Department of Nephrology, Zhangqiu People’s Hospital, No.1920, Zhangqiu MingShui huiquan road, Jinan, China

**Keywords:** Volume load, Cardiovascular disease, Bioelectrical impedance analysis, Body fluid composition

## Abstract

**Background:**

Previous studies indicated that the clustering of major cardiovascular disease (CVD) risk factors is common, and multiple unhealthy lifestyles are responsible for the clustering of CVD risk factors. However, little is known about the direct association between the volume load and the clustering of CVD risk factors in general population.

**Methods:**

We investigated the association of the clustering of CVD risk factors (defined as two or more of the following factors: hypertension, diabetes, dyslipidemia and overweight) with volume load, which was evaluated by bioelectrical impedance analysis. Hypovolaemia was defined as extracellular water/total body water (ECW/TBW) at and under the 10th percentile for the normal population.

**Results:**

Among the 7900 adults, only 29.3% were free of any pre-defined CVD risk factors and 40.8% had clustering of CVD risk factors. Hypovolaemia in clustering group was statistically higher than that either in the single or in the none risk factor group, which was 23.7% vs. 17.0% and 10.0%, respectively (P <0.001). As a categorical outcome, the percentage of the lowest quartiles of ECW/TBW and TBW/TBWwatson in clustering group were statistically higher than either those in the single or in the none risk factor group, which were 44.9% vs. 36.9% and 25.1% (P <0.001), 36.2% vs. 32.2% and 25.0%, respectively (P <0.001). After adjusting of potential confounders, hypovolaemia was significantly associated with clustering of CVD risk factors, with an OR of 1.66 (95% CI, 1.45-1.90).

**Conclusions:**

Hypovolaemia was associated with clustering of major CVD risk factors, which further confirms the importance of lifestyle for the development of CVD.

## Background

Cardiovascular disease (CVD) is the main cause of mortality and morbidity worldwide [1–3]. Hypertension, diabetes, dyslipidemia and overweight are the major risk factors of CVD [4–6]. In China, the prevalence of clustering of major CVD risk factors was 36.2%, only 31.1% were free of any pre-defined CVD risk factors [7].

Multiple unhealthy lifestyles, including habitual drinking, physical inactivity and use of NSAIDs, are responsible for clustering of CVD risk factors [7]. In addition, lifestyle interventions e.g., physical exercise and consuming a low-fat diet, may effectively prevent the development of type 2 diabetes, hypertension and dyslipidemia in high-risk subjects [8–10]. Therefore, comprehensive lifestyle interventions may be an effective strategy to control CVD risk factors and reduce the burden of CVD. Methods for screening the risk of CVD is a fundamental strategy for the primary prevention of CVD, but the patients with the highest risks should be identified in order to maximize the benefit/cost ratio of treatments.

Clustering of CVD risk factors was positively associated with Chronic kidney disease (CKD) [11]. Recent observations indicated that higher levels of water intake were associated with slower progression of CKD [12, 13]. Epidemiologic evidence suggests that the balance of water intake and output may have implications for development of CKD. Hypovolaemia caused by arduous physical labor or high ambient temperaturemay be associated with CKD [14, 15]. Arginine vasopressin (AVP), a crucial peptide hormone that regulates water homeostasis, may contribute to CVD progression In a rat model of 5/6 nephrectomy, increased water intake decreases AVP and reduces histological damage [16, 17]. However, little is known about the direct association between volume load and clustering of CVD risk factors in general population. Therefore, we performed a cross-sectional study on a large scale population to examine the relationship between volume load and clustering of CVD risk factors, which was evaluated based on bioelectrical impedance analysis.

## Methods

### Study population

A total of 7900 adults who visited the Health Checkup Clinic consecutively in Qianfoshan Hospital of Shandong University were enrolled in the study. This study excludes outpatient or clinical patients. Patients with acute kidney injury, amputation, heart failure, severe liver disease, infection disease, malignant disease and pregnancy were excluded. Patients on diuretic drugs or any kind of renal replacement therapy were also excluded. The investigation was started in July 2010 and ended in December 2013. The ethics committee of Qianfoshan Hospital approved the study. All participants were given written informed consent prior to data collection.

### Blood biochemistry measurements and biometric parameters

Blood was collected by means of venipuncture after an overnight fast of at least 10 hours. Serum creatinine was measured by the Roche enzymatic method using an automatic biochemistry analyzer (Roche P Modular with Roche Creatininase Plus assy, Hoffman-La Roche, Ltd., http://www.roche.com). Glomerular filtration rate (eGFR) was estimated using the Chronic Kidney Disease Epidemiology Collaboration (CKD-EPI) equation [18, 19]. Decreased renal function was defined as the eGFR below 60 ml/min/1.73 m^2^. Hemoglobin, fasting blood glucose, serum uric acid, serum total cholesterol (TC), low-density lipoprotein cholesterol (LDL-C), and triglycerides (TG) were also measured by automatic biochemistry analyzer.

Sociodemographic characteristics, health history (eg, hypertension, diabetes), and lifestyle behaviors were obtained through questionnaire. The body mass index (BMI) was calculated as weight (in kilograms) divided by height squared (in square meters). Blood pressure was measured using a sphygmomanometer, and three measurements were taken at 5 min intervals. The mean of the three readings was calculated, unless the difference between the readings was greater than 10 mmHg, in which case the mean of the two closest measurements was used. All of the investigators have completed a training program of the methods and procedures of the study.

### Body fluid composition

Below are our methods to assess the components of body fluid composition. A multi-frequency bioelectrical impedance analysis (BIA) device (Body Composition Monitor, BMC, Fresenius Medical Care D GmbH), which measures 50 different frequencies 5 to 1000 kHz was used. Input variables include the patient’s height, weight, age, and sex. The measurements were performed after a 20-min resting period with the patient in the supine position. The procedure was performed for patients who had not consumed a heavy meal in at least 4 to 5 hours, or exercised 12 hours before the test, or consumed alcohol or caffeine 24 hours before the test. Electrodes were attached to the hand and foot on the nondominant side of the body, after the patient had been in recumbent position for 5 min. The following parameters were collected: extracellure water (ECW), intracellular water (ICW), total body water (TBW), ECW/TBW. ECW was standardized by height (NECW). Anthropometric formulas with tracer dilution techniques (e g., Waston) have been widely used to calculate TBWwatson [20]. TBWwatson in normal healthy subjects (Watson’s formula): Male, 2.447 + (0.09156 × age) + (0.1074 × height) + (0.3362 × weight); Female:-2.097 + (0.1069 × height) + (0.3362 × weight); Dry mass index (DMI) = [(weight-TBW)/height^2^)]; Lean body mass (LBM), which can be calculated using the formula (TBW/0.733) in BIA, are commonly used for accessing nutritional status [21].

### Determination of arterial stiffness

cfPWV was assessed using the SphygmoCor device (AtCor Medical LtD., Sydney, Australia) as previously described [22]. A measuring tape was used to assess the distance between the carotid and femoral artery recording sites. cfPWV was calculated automatically by dividing this distance by the time interval between the rapid upstroke in the pulse wave at the carotid and femoral arteries using the peak of the R-wave on electrocardiography as a reference point.

### Assessment criteria

We investigated the clustering of four major CVD risk factors, defined as two or more of the following: hypertension, diabetes, dyslipidemia and overweight. Hypertension was defined by the finding on three consecutive measurements at the clinic obtained two weeks apart of a mean systolic blood pressure of more than 140 mm Hg or a mean diastolic blood pressure of more than 90 mm Hg, or both, or patients already being priscribed by antihypertensive medicaments. Diabetes was defined as fasting blood glucose ≥7.0 mmol/L or the use of hypoglycemic agents or self-reported history of diabetes. Dyslipidemia was defined by the presence of at least one of the following: serum TC level ≥5.2 mmol/L, TG level ≥1.7 mmol/L, or LDL-C level ≥3.4 mmol/L [23]. Overweight was defined as a BMI ≥24 kg/m^2^ [24]. Hypovolaemia was defined as extracellular water/total body water (ECW/TBW) at and under the 10th percentile for the normal population (ECW/TBW ≤ 0.3167). As a categorical outcome, we calculated both the lowest and highest quartiles of ECW/TBW (0.3229 and 0.3357) and TBW/TBWwatson (0.8421 and 0.9008) for the normal population to describe the distribution of ECW/TBW and TBW/TBWwatson in different CVD risk factor groups.

### Statistical analysis

Data were presented as proportions for categorical variables and mean ± SD for continuous variables. The significance of differences in continuous variables between groups were tested using *t* - test or one-way analysis of variables. The difference in the distribution of categorical variables was determined by Chi-square test. The association between hypovolaemia and clustering of CVD risk factors was analyzed using logistic regression models. Age- and Sex-adjusted adds ratios (ORs) with 95% confidence interval (CI) were reported. We then used forward selection method and built a parsimonious model to adjust for other confounders. Covariates under consideration include age (continuous), sex (female vs. male), hypovolaemia, hemoglobin (continuous), eGFR (decreased or not ) and serum uric acid (continuous). We forced age and gender into the model.

All analyses were performed by SPSS statistical package, version 16.0 (SPSS, Inc., Chicago, IL). A *P* value of less than 0.05 is considered statistically significant.

## Results

The baseline clinical characteristics of participants were presented in Table 1. A total of 7900 individuals (5467males, mean age 38.8 ± 8.5 and 2433 females, mean age 37.4 ± 7.3) were enrolled in the study following the inclusion criteria. More than one half (52.6%) males and 14.0% females had clustering of CVD risk factors. The body fluid composition was obtained by the BIA and anthropometric formula. TBW value was 43.3 ± 5.1 (L) for males, and 30.7 ± 3.4 (L) for females. The average values of TBW were lower than those of TBWwatson. In contrast, the values of the TBW/TBWwaston ration were <1 in both males and females, indicating a fluid volume deficit.

**Table 1:** **Baseline clinical characteristics of participants**

	Male	Female	P
Number (n, %)	5467 (69.2)	2433 (30.8)	/
Age (years)	38.8 ± 8.5	37.4 ± 7.3	< 0.001
Height (cm)	172.7 ± 6.0	161.1 ± 5.3	< 0.001
BW (kg)	76.9 ± 11.6	58.8 ± 8.6	< 0.001
BMI ( kg/m^2^)	25.8 ± 3.5	22.7 ± 3.1	< 0.001
Clinical findings			
SBP (mmHg)	131.7 ± 15.7	118.6 ± 13.8	< 0.001
DBP (mmHg)	78.5 ± 12.2	70.9 ± 10.0	< 0.001
Hypertension (n,%)	1369 (25.2)	169 (7.0)	< 0.001
Diabetes (n, %)	336 (6.2)	31(1.3)	< 0.001
Overweight (n, %)	3714 (68.8)	710 (29.6)	< 0.001
Dyslipidemia (n, %)	2965 (55.0)	608 (25.5)	< 0.001
HBsAg (n, %)	168 (3.5)	42 (2.0)	0.001
Serum uric acid (μmol/L)	353.1 ± 82.7	220.4 ± 58.8	< 0.001
Blood glucose (mmol/L)	5.6 ± 1.2	5.2 ± 0.8	< 0.001
Hemoglobin (g/L)	151.6 ± 9.2	126.5 ± 12.0	< 0.001
Hct %	44.2 ± 2.6	37.5 ± 3.1	< 0.001
eGFR (ml/min/1.73 m^2^)	105.1 ± 12.6	104.1 ± 14.1	0.002
Body fluid composition			
TBW (L)	43.3 ± 5.1	30.7 ± 3.4	< 0.001
ICW (L)	29.2 ± 3.5	20.6 ± 2.3	< 0.001
ECW (L)	14.1 ± 1.8	10.1 ± 1.1	< 0.001
NECW (L/m))	8.1 ± 0.9	6.3 ± 0.6	< 0.001
LBM (kg)	59.1 ± 7.0	41.9 ± 4.6	< 0.001
ECW/TBW	0.32 ± 0.01	0.33 ± 0.01	< 0.001
TBW waston (L)	50.4 ± 4.3	34.9 ± 3.1	< 0.001
TBW/TBW watson	0.86 ± 0.05	0.88 ± 0.04	0.001
DMI (kg/m^2^)	11.3 ± 2.4	10.8 ± 2.3	< 0.001
CVD risk factor (n, %)			< 0.001
None	969 (18.3)	1269 (54.1)	/
Single	1547 (29.2)	746 (31.8)	/
Cluster	2791 (52.6)	329 (14.0)	/
Arterial stiffness			
cfPWV (cm/s)	1379.2 ± 171.1	1256.0 ± 139.5	< 0.001

Abbreviations: BW, body weight; BMI, body mass index; SBP, systolic blood pressure; DBP, diastolic blood pressure; Hb, hemoglobin; Hct, hematocrit; eGFR, estimated glomerular filtration rate; CVD, cardiovascular disease; TBW, total body water; ICW, intracellular water; ECW, extracellular water; NECW, normalization extracellular water; LBM, lean body mass; DMI, dry mass index; cfPWV, carotid-femoral pulse wave velocity.

**Figure 1:**
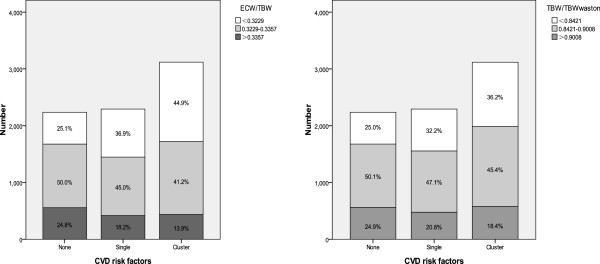
**The distribution of ECW/TBW and TBW/TBW watson in different CVD risk factor groups.**

Age, BW, BMI, TBW, ICW, ECW, NECW, LBM, TBWwaston and DMI were statistically higher in clustering group than either in the single or in the none risk factor group (P <0.001). However, the ratio of ECW/TBW and TBW/TBWwatson were lower in clustering group than other two groups (P <0.001). Hypovolaemia in clustering group (23.7%) were statistically higher than either in the single or in the none risk factor group, which 17.0% and 10.0%, respectively (P <0.001). cfPWV (1419.0 ± 172.5) were statistically higher in clustering group than either in the single or in the none risk factor group, which were 1308.4 ± 139.2 and 1245.5 ± 144.1 cm/s, respectively, P <0.001, Table 2. As a categorical outcome, the percentage of the lowest quartiles of ECW/TBW and TBW/TBWwatson in clustering group were statistically higher than either in the single or in the none risk factor group, which was 44.9% vs. 36.9% and 25.1%, 36.2% vs. 32.2% and 25.0%, respectively (P <0.001), Figure 1.

**Table 2:** **Body fluid composition according to CVD risk factors**

	None	Single	Cluster	P
Prevalence (%)	29.3	30.0	40.8	/
Age (year)	35.1 ± 7.8	38.3 ± 8.1	41.0 ± 7.6	< 0.001
Male (n,%)	969 (43.3)	1547 (67.5)	2791 (89.5)	< 0.001
BW (kg)	59.3 ± 8.1	70.8 ± 11.4	80.5 ± 11.1	< 0.001
BMI (kg/m^2^)	21.3 ± 1.8	24.7 ± 3.0	27.5 ± 2.9	< 0.001
TBW (L)	34.0 ± 6.0	39.1 ± 6.8	43.6 ± 6.1	< 0.001
ICW (L)	22.8 ± 4.1	26.3 ± 4.6	29.4 ± 4.1	< 0.001
ECW (L)	11.2 ± 2.0	12.7 ± 2.2	14.2 ± 2.1	< 0.001
NECW (L/m))	6.7 ± 0.9	7.5 ± 1.0	8.2 ± 0.9	< 0.001
LBM (kg)	46.3 ± 8.2	53.3 ± 9.3	59.5 ± 8.3	< 0.001
ECW/TBW	0.33 ± 0.01	0.33 ± 0.01	0.32 ± 0.01	< 0.001
Hypovolaemia (n,%)	223 (10.0)	397 (17.0)	738 (23.7)	< 0.001
TBWwatson (L)	39.0 ± 6.8	45.2 ± 7.3	50.7 ± 5.8	< 0.001
TBW/TBWwatson	0.87 ± 0.05	0.86 ± 0.05	0.86 ± 0.05	< 0.001
DMI ( kg/m^2^)	9.1 ± 1.4	11.1 ± 2.2	12.6 ± 2.1	< 0.001
cfPWV (cm/s)	1245.5 ± 144.1	1308.4 ± 139.2	1419.0 ± 172.5	< 0.001

Abbreviations: CVD, cardiovascular disease; BW, body weight; BMI, body mass index; TBW, total body water; ICW, intracellular water; ECW, extracellular water; NECW, normalization extracellular water; LBM, lean body mass; DMI, dry mass index; cfPWV, carotid-femoral pulse wave velocity.

After adjusted for potential confounders, age, hemoglobin, serum uric acid and hypovolaemia were associated with clustering of CVD risk factors, with ORs of 1.08 (95% CI, 1.07-1.09), 1.04 (95% CI, 1.04-1.05), 1.007 (95% CI, 1.006-1.007), 1.66 (95% CI, 1.45-1.90), (Table 3).

**Table 3:** **Multivariate logistic regression analysis for association of clustering of CVD risk factors with different variables**

Variables	Age- and Sex-adjusted OR ^a^(95% CI)	P	Multivariable adjusted OR ^b^(95% CI)	P
Age	1.07 (1.06-1.08)	< 0.001	1.08 (1.07-1.09)	< 0.001
Sex	0.15 (0.13-0.17)	< 0.001	0.92 (0.75-1.12)	0.40
Hemoglobin	1.04 (1.04-1.05)	< 0.001	1.04 (1.04-1.05)	< 0.001
Serum uric acid	1.007 (1.006-1.008)	< 0.001	1.007 (1.006-1.007)	< 0.001
Decreased eGFR	1.63 (0.31-8.62)	0.56	1.64 (0.29-9.2)	0.57
Hypovolaemia	1.84 (1.62-2.1)	< 0.001	1.66 (1.45-1.90)	< 0.001

Note: The regression model only included the none and the clustering groups.

^a^Except for OR of age and sex, all ORs were age and sex adjusted.

^b^Model was adjusted for age, sex, hemoglobin, serum uric acid, decreased eGFR and hypovolaemia.

## Discussion

Our study revealed a high prevalence of clustering of CVD risk factors in the adult population. Among the total participants, only 29.3% were free of any pre-defined CVD risk factors and 40.8% had clustering of CVD risk factors. The epidemiological studies have demonstrated that CVD risk factors could cluster in twins and among coronary prone family members [25, 26], suggesting that genetic factors might play an important role in the development of CVD risk factors. of these CVD risk factors, hypertension and diabetes mellitus are multifactorial disease under the influence of environmental factors [27].

Our study showed that age, blood uric acid, and hemoglobin were associated with the clustering of CVD risk factors. Many studies suggested that high levels of uric acid are independent risk factors of CVD [28, 29]. In general population, increased level of hemoglobin is associated with atherosclerosis [30], while the mechanism is unknown. Studies have shown that hemoglobin was associated with the ratio of triglyceride/cholesterol [31], a risk factor for atherosclerosis. Furthermore, the elevated hemoglobin may increase blood viscosity and injury the vascular endothelium.

A multi-frequency bioelectrical impedance analysis (BIA) device is useful for assessing fluid and nutritional status in a noninvasive and easily accessible manner. We assessed fluid volume status in the population using the ECW/TBW and the TBW/TBWwatson ratio, which is calculated using the Watson formula adjusted for height, weight, age, and gender. Importantly, our study demonstrated that hypovolaemia was associated with clustering of major CVD risk factors. hypovolaemia might be a result of insufficient fluid intake. It has already been shown that there is a common hormone, angiotensin contributing to three of major diseases today, obesity, diabetes and hypertension [32]. Yet, this hormone is released under normal physiological conditions of hypovolaemia, which also stimulates the release of aldosterone. The presence of angiotensin and aldosterone is associated with metabolic dysfunction through increased insulin resistance, decreased glucose transport in skeletal muscle cells and vascular smooth muscle cells as well as decreased cardiac Na^+^K^+^ATPase activity [33]. Furthermore, increased water intakecould decreased the insulin resistance in animal models of obesity [34]. On another hand, it has been suggested that hypovolaemia increased the activity of the sympathetic nervous system [35]. Therefore, hypovolaemia induced reduction of cell volume causes insulin resistance, decreased glucose transport, and cellular metabolic dysfunction [36].

Our study has limitations that deserve mention. First, it was implemented on a voluntary bias and was not based on a community-based screening. Selecting bias in the study limited the extension of the results from this study. The between-individual variation exists when using ECW/TBW to define volume status. Second, non-traditional risk factors of clustering of CVD risk factors were not investigated in the present study. This study excluded patients who were taking diuretics. These patients are likely to have hypertension or diabetes, therefore the prevalence of clustering CV risks may be underestimated. The association between fluid status and clustering of CVD risks in the diuretics taking group needs further investigations. Third, our data was cross-sectional and do not provide an insight into the mechanisms responsible for the observed associations.

## Conclusions

Our study indicates that clustering of CVD risk factors is common and positively associated with hypovolaemia in adult population. Future clinical trials need to be implemented to confirm whether increased consumption of fluids would be feasible for controlling the clustering of CVD risk factors.
